# Sleep time on back as a predictor of adherence to positive airway pressure therapy

**DOI:** 10.1038/s41598-023-49959-5

**Published:** 2023-12-16

**Authors:** Hyun-Ho Kwak, Ji-Hwan Park, Dong-Joo Lee, Gyo Han Bae, Sung-Dong Kim, Kyu-Sup Cho

**Affiliations:** Department of Otorhinolaryngology and Biomedical Research Institute, Pusan National University School of Medicine, Pusan National University Hospital, Busan, South Korea

**Keywords:** Diseases, Medical research

## Abstract

Upper airway collapse can be effectively dealt with positive airway pressure (PAP), and patient adherence is considered as a major determining factor for success of PAP therapy. This study was performed to determine the potential factors affecting the adherence to PAP in patients with OSA by using polysomnography (PSG) parameters recorded for diagnosis of OSA. The data of 158 patients between December 2018 and July 2021 were collected. They were prescribed with PAP and used the device during the adaptation period for 90 days. They were categorized into adherent and non-adherent group according to the criteria of good adherence as use of PAP ≥ 4 h per night on 70% of nights. Demographic, clinical characteristics, and PSG results were reviewed. Among 158 patients engaged in PAP therapy, 121 patients (76.6%) met the criteria of good adherence. No significant differences were found in good adherence rate regarding demographic and clinical characteristics. None of the polysomnographic factors showed significant differences between adherent and non-adherent groups. However, the percentage of sleep time on back in the adherent group was significantly higher than non-adherent group (p = 0.041). The cut-off value was determined to be 41.45% (95% confidence interval 0.43 to 0.79) by receiver operating characteristic curve analysis and the odds ratio was calculated as 2.97. Only the percentage of sleep time on back appeared to be polysomnographic predictor for identifying good adherence to PAP therapy in OSA patients. However, the conclusions may be limited in generalization due to the small sample size.

## Introduction

The most common type of sleep-related breathing disorder is obstructive sleep apnea (OSA). OSA can partially or completely obstruct the upper airway, and recurrent obstructive events may lead to nocturnal hypoxemia, sleep fragmentation, and excessive daytime somnolence^1^. In addition, OSA is known to have association with various cardiovascular complications such as hypertension, pulmonary hypertension or cardiac arrest associated with heart failure, myocardial infarction, and stroke^2–4^.

Overnight polysomnography (PSG) is essential to diagnose OSA and it is also needed when titrating positive airway pressure (PAP)^5^. PAP has been the most effective and widely used therapy for treating moderate and severe OSA^6,7^. PAP maintains the patency of upper airway and helps to reduce the work of breathing and improve alveolar ventilation as well as hypoxemia^8^. The effect of PAP largely depends on the willingness of the patient to use the device during sleep since it is a self-administered therapy^9^. Therefore, adherence to the therapy should be considered as a major determinant for success of PAP therapy^10^.

Adherence rates, in general, range from 30 to 60%, even though there have been numerous developments in the device itself including quieter pumps, softer masks, and improved portability^10,11^. As adherence to PAP remains a clinically significant issue, various studies have revealed the possible factors that might have an influence on the adherence to PAP. Patient’s complaints related to PAP use, such as inconvenience, poor mask fit, mask discomfort, nasal complications, frequent awakening, feelings of claustrophobia, and aversion to PAP treatment could affect PAP adherence^12,13^. Many clinical parameters as well as psychological factors have been investigated to predict compliance, but the reported results of the studies have been inconsistent^14,15^. Furthermore, polysomnographic predictors of good adherence to PAP therapy remain under investigation in OSA patients. The purpose of this study was to evaluate the potential factors affecting the adherence to PAP in patients with OSA by using PSG parameters recorded for diagnosis of OSA.

## Methods

We retrospectively investigated the data of 158 OSA patients who underwent PAP therapy from a single center. The prescription date of PAP ranged from December 2018 to July 2020. The Institutional Review Board of Pusan National University Hospital has reviewed and approved the study protocol (H-2209-025-119) as well as the waiver of the informed consent requirement considering the retrospective study design involving anonymized data. This study was conducted according to the ethical standards of the 1964 Declaration of Helsinki and its later amendments.

Full-night PSG (Embla N7000, Embla Systems, Broomfield, CO) was performed in all patients before PAP treatment. The patients who were diagnosed with OSA were included in the review. They had apnea–hypopnea index (AHI) of five or more, and must have suffered from at least one of the clinical symptoms, for instance, snoring, sleep apnea, morning headache, tiredness, or daytime sleepiness. Patients who had factors which might affect the adherence rate were excluded from the study, for instance, patients with severely deviated nasal septum and severe nasal obstruction, patients who were suffering from rhinosinusitis or allergic rhinitis, patients who had undergone surgery or who had severe cardiopulmonary disease. The severity of disease was classified according to AHI (mild, 5 ≤ AHI < 15; moderate, 15 ≤ AHI < 30; and severe, AHI ≥ 30). The body position was measured using a position sensor strapped on the chest with thoracic band approximately in the midline. This sensor can detect whether the position is supine, prone, right decubitus or left decubitus. The patient was continuously monitored and video recorded throughout the PSG by registered polysomnographic technician. Some misidentified body positions were corrected in real time or after the examination by reviewing the PSG and video record.

The patients underwent PAP therapy for initial 90 days, which was designated as adaptation period. The 90 days of adaptation period is under coverage of Korean National Health Insurance (NHI). If the patients used the PAP device regularly and showed good adherence, they could use the device under insurance coverage after the adaptation period. With poor adherence, they cannot get insurance for using PAP device after adaptation period. The criteria for good adherence was defined as using a PAP device regularly for more than 4 h per night for > 70% of the recorded period. The doctor and the device manager tried to enhance the PAP adherence. They monitored the patients closely and intervened by rapidly addressing the problems such as air leakage or discomfort from masks or skin irritation while using the PAP device. We categorized all patients into adherent and nonadherent users based on the data retrieved from their PAP devices.

Demographic, clinical features, and pretreatment PSG results were reviewed. Sex, age, body mass index (BMI, kg/m^2^), medical history, neck and waist circumferences (cm), and scores of Epworth Sleepiness Scale (ESS) were collected for demographic and clinical analysis. Pretreatment PSG variables included total sleep time (TST), percentage of sleep time on back (supine sleep time), sleep efficiency, arousal index, percentage of time spent in N1, N2, N3, REM, apnea index (AI), total AHI, the proportion of apnea and hypopnea events in supine position from total apnea and hypopnea events, AHI in supine position, AHI in lateral position, positional OSA (supine/non-supine AHI ratio ≥ 2), mean arterial oxygen saturation (SaO_2_), lowest SaO_2_, and cumulative time percentage with SaO_2_ < 90% (CT90).

Continuous variables were presented as mean (standard deviation [SD]) or median (interquartile range [IQR]) for normally and non-normally distributed data, respectively. Categorical variables were expressed as a number (percentage). Statistical significance was assessed using independent *t*-test or Wilcoxon rank-sum test for continuous variables and Chi-square test or Fisher’s exact test for categorical variables. Receiver operating characteristic (ROC) curve analyses were used to determine the cut-off value. Univariate and multivariate analysis by logistic regression were conducted to control the covariates or confounders and to identify predictive factors for adherence rate. Statistical analyses were performed using R statistical software (v4.1.3; R Core Team, 2021). The result was considered to be statistically significant when the p-value was less than 0.05.

## Results

In the study period, 158 OSA patients who were diagnosed by PSG were engaged in PAP therapy. The 131 (82.9%) males and 27 (17.1%) females had an age range of 21 to 88 years and a mean age of 53.2 years. The mean BMI was 27.1 ± 4.8 kg/m^2^ (range 17.0–46.7 kg/m^2^). Sixty-two patients (39.2%) had medical history of hypertension, 19 (12.0%) patients had cardiac disease, 12 (7.6%) had neurovascular disease, and 19 (12.0%) had diabetes. Mean neck circumference and median waist circumference were 38.4 ± 3.9 cm and 93.0 cm (IQR 85.0–98.8 cm), respectively. Mean ESS score was 8.1 ± 4.9 (Table 1).

**Table 1:** Demographic and clinical characteristics.

Characteristics	Values (n = 158)
Sex	
Male	131 (82.9)
Female	27 (17.1)
Age, years	53.2 ± 13.5
BMI, kg/m^2^	27.1 ± 4.8
Hypertension	62 (39.2)
Cardiac disease	19 (12.0)
Neurovascular disease	12 (7.6)
Diabetes	19 (12.0)
Neck circumference, cm	38.4 ± 3.9
Waist circumference, cm	93.0 (85.0–98.8)
ESS	8.1 ± 4.9

Data are expressed as the number (percentage) except age, BMI, neck circumference, ESS (mean ± standard deviation) and waist circumference (median and interquartile range).

*BMI* body mass index, *ESS* Epworth sleepiness scale.

### Comparison of demographic and clinical characteristics between adherent group and non-adherent group

One hundred twenty-one patients (76.6%) met the criteria of good adherence during the adaptation period for 90 days. The mean percentage of days on which the use of PAP exceeded 4 h per night during the adaptation period were 84.8 ± 11.1% in the adherent group and 31.5 ± 26.4% in the non-adherent group. There were no significant differences between the two groups regarding sex, age, BMI, history of hypertension, cardiac disease, neurovascular disease, diabetes, neck circumference, waist circumference, and ESS (Table 2).

**Table 2:** Demographic and clinical characteristics of adherent and non-adherent groups.

Characteristics		Adherent (n = 121)	Non-adherent (n = 37)	*p*-value
Sex				0.930
Male		101 (83.5)	30 (81.1)	
Female		20 (16.5)	7 (18.9)	
Age, years		53.5 ± 12.7	52.2 ± 16.1	0.667
BMI, kg/m^2^		27.2 ± 4.6	26.6 ± 5.7	0.538
Hypertension	−	73 (60.3)	23 (62.2)	0.994
	+	48 (39.7)	14 (37.8)	
Cardiac disease	−	106 (87.6)	33 (89.2)	1.000
	+	15 (12.4)	4 (10.8)	
Neurovascular disease	−	114 (94.2)	32 (86.5)	0.154
	+	7 (5.8)	5 (13.5)	
Diabetes	−	105 (86.8)	34 (91.9)	0.567
	+	16 (13.2)	3 (8.1)	
Neck circumference, cm		38.6 ± 3.8	37.8 ± 4.0	0.290
Waist circumference, cm		93.0 (85.0–98.0)	90.0 (82.5–100.0)	0.593
ESS		8.0 ± 4.9	8.7 ± 5.1	0.449

Data are expressed as the number (percentage) except age, BMI, neck circumference, ESS (mean ± standard deviation) and waist circumference (median and interquartile range).

*BMI* body mass index, *ESS* Epworth sleepiness scale.

### Comparison of polysomnographic variables between adherent group and non-adherent group

There was no significant correlation with good adherence for TST, sleep efficiency, arousal index, percentage of sleep stages including N1, N2, N3, and REM, total AI, total AHI, proportion of apnea and hypopnea in supine position, supine AHI, lateral AHI, positional OSA, mean SaO_2_, lowest SaO_2_, and CT90. However, the percentage of sleep time on back over TST was 69.0% in the adherent group and 56.2% in the non-adherent group, which was statistically different (*p* = 0.041) (Table 3).

**Table 3:** Polysomnographic parameters of adherent and non-adherent groups.

Variables	Overall	Adherent (n = 121)	Non-adherent (n = 37)	*p*-value*
Total sleep time, min	322.9 ± 61.6	325.8 ± 62.8	313.7 ± 57.3	0.276
Sleep time on back, %	67.5 (40.8–87.7)	69.0 (44.6–91.3)	56.2 (31.8–79.4)	0.041
Sleep efficiency, %	80.6 ± 13.7	80.7 ± 13.4	80.2 ± 14.6	0.838
Arousal index/h	31.1 ± 18.4	31.9 ± 19.1	28.6 ± 15.8	0.293
Stage 1, % of TST	30.3 ± 16.1	31.1 ± 16.6	27.6 ± 14.0	0.214
Stage 2, % of TST	50.7 ± 13.3	50.1 ± 13.5	53.0 ± 12.5	0.222
Stage 3, % of TST	6.5 ± 8.0	6.3 ± 8.0	7.4 ± 7.9	0.434
REM, % of TST	12.4 ± 6.3	12.5 ± 6.2	12.0 ± 6.6	0.667
Total AI/h	27.6 ± 24.2	29.1 ± 24.5	22.9 ± 23.0	0.166
Total AHI/h	45.4 ± 24.4	46.9 ± 24.1	40.7 ± 25.4	0.197
Apnea–hypopnea on back ratio, %	76.9 ± 25.4	78.4 ± 24.5	72.1 ± 28.1	0.224
Supine AHI/h	55.3 ± 25.1	55.8 ± 25.1	53.8 ± 25.6	0.673
Lateral AHI/h	24.3 ± 25.7	24.5 ± 25.6	23.8 ± 26.4	0.897
Supine/non-supine AHI ≥ 2	106 (67.1)	80 (66.1)	26 (70.3)	0.787
Mean SaO_2_, %	94.2 (92.3–95.5)	94.2 (92.5–95.6)	94.1 (92.3–95.5)	0.849
Lowest SaO_2_, %	78.0 ± 8.4	77.5 ± 8.7	79.7 ± 7.2	0.133
CT90, %	5.1 (1.1–21.4)	4.9 (1.1–21.3)	6.6 (1.4–21.6)	0.778

Data are expressed as the mean ± standard deviation except sleep time on back, mean SaO_2_, CT90 (median and interquartile range) and supine/non-supine AHI ≥ 2 (number and percentage).

*AHI* apnea–hypopnea index, *AI* apnea index, *CT90* cumulative time percentage with SaO_2_ < 90%, *REM* rapid eye movement, *SaO*_2_ arterial oxygen saturation, *TST* total sleep time.

p-values were calculated with adherent and non-adherent group.

### PAP adherence according to the cut-off value of sleep time on back

**Figure 1:**
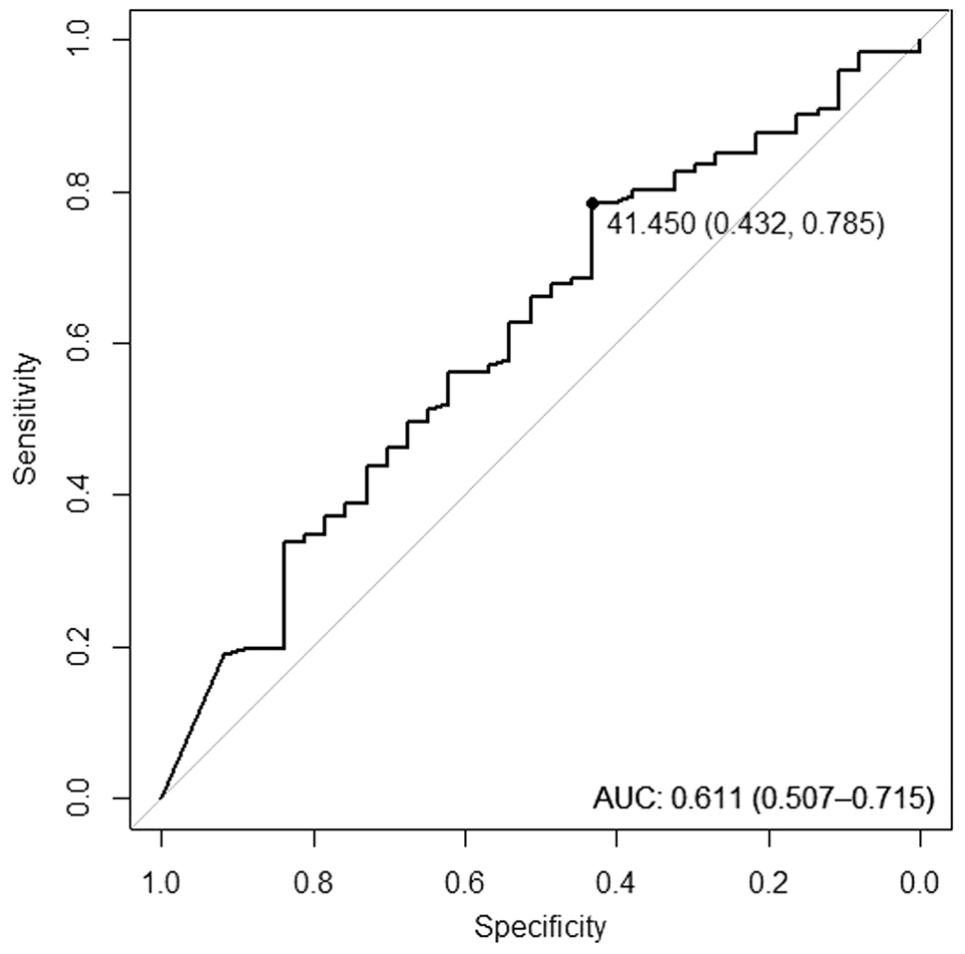
Receiver-operating characteristic (ROC) curve for relationship between the percentage of sleep time on back and PAP adherence. Cut-off value with specificity and sensitivity in parentheses are given in the figure. Area under the ROC curve is 0.61 with 95% confidence interval of 0.51–0.72.

The optimal cut-off value for percentage of sleep time on back in predicting adherence to PAP therapy was determined by receiver operating characteristic (ROC) curve. The cut-off value was 41.45% (95% confidence interval (CI) 0.43 to 0.79) with sensitivity, specificity, positive predictive value (PPV), negative predictive value (NPV) of 0.79, 0.43, 0.82, and 0.38, respectively. The area under the ROC curve (AUC) was 0.61 (95% CI 0.51 to 0.72) (Fig. 1).

PAP adherence between adherent group and non-adherent group according to the cut-off value of sleep time on back was analyzed. The PAP adherence rate in patients with percentage of sleep time on back of 41.45% or more was 78.5% (95/121) and that in patients with less than 41.45% was 21.5% (26/121), which showed statistically significant difference (*p* = 0.016) (Table 4).

**Table 4:** Adherence rate according to cut-off value of sleep time on back.

Sleep time on back	Adherent (n = 121)	Non-adherent (n = 37)	*p*-value
≥ 41.45%	95 (78.5)	21 (56.8)	0.016
< 41.45%	26 (21.5)	16 (43.2)	

Data are expressed as the number (percentage).

The univariate analysis included all the variables from demographic data and polysomnographic data that were shown in Tables 2 and 3. It indicated that the adherence rate was significantly associated with the percentage of sleep time on back and especially the percentage of sleep time on back of 41.45% or more (OR 1.01; 95% CI 1.00–1.02; *p* = 0.047, OR 2.78; 95% CI 1.27–6.08; *p* = 0.010, respectively). After excluding all the variables with multicollinearity and including potential confounders such as gender, age, BMI, comorbidities of hypertension, cardiac disease or diabetes, irrespective of statistical significance, the percentage of sleep time on back of 41.45% or more was still significantly associated with the adherence rate in the multivariate analysis (OR 2.97; 95% CI 1.31–6.77; *p* = 0.009) (Table 5).

**Table 5:** Univariate and multivariate analysis of factors related to adherence rate.

Variables	Univariate analysis		Multivariate analysis	
	OR (95% CI)	*p*-value	OR (95% CI)	*p*-value
Female	0.85 (0.33–2.20)	0.736	0.76 (0.27–2.11)	0.598
Age	1.01 (0.98–1.04)	0.622	1.03 (0.99–2.11)	0.146
BMI	1.03 (0.95–1.12)	0.485	1.03 (0.94–1.06)	0.494
Hypertension	1.08 (0.51–2.30)	0.842	1.03 (0.94–1.13)	0.828
Cardiac disease	1.17 (0.36–3.76)	0.795	0.91 (0.37–2.20)	0.914
Neurovascular disease	0.39 (0.12–1.32)	0.131	0.93 (0.25–3.47)	0.137
Diabetes	1.73 (0.47–6.29)	0.407	0.93 (0.25–3.47)	0.717
Neck circumference	1.06 (0.96–1.17)	0.278		
Waist circumference	1.00 (0.97–1.03)	0.859		
ESS	0.97 (0.90–1.05)	0.435		
Total sleep time, min	1.00 (1.00–1.01)	0.296		
Sleep time on back, %	1.01 (1.00–1.02)	0.047		
Sleep time on back ≥ 41.45%	2.78 (1.27–6.08)	0.010	2.97 (1.31–6.77)	0.009
Sleep efficiency	1.00 (0.97–1.03)	0.829		
Arousal index	1.01 (0.99–1.03)	0.339		
Stage 1, % of TST	1.02 (0.99–1.04)	0.253		
Stage 2, % of TST	0.98 (0.95–1.01)	0.239		
Stage 3, % of TST	0.98 (0.94–1.03)	0.434		
REM, % of TST	1.01 (0.96–1.08)	0.652		
Total AI	1.01 (1.00–1.03)	0.179		
Total AHI	1.01 (1.00–1.03)	0.181		
Apnea–hypopnea on back ratio	1.01 (1.00–1.02)	0.189		
Supine AHI	1.00 (0.99–1.02)	0.666		
Lateral AHI	1.00 (0.99–1.02)	0.894		
Supine/Non-supine AHI ≥ 2	0.83 (0.37–1.84)	0.638		
Mean SaO_2_	1.03 (0.93–1.15)	0.530		
Lowest SaO_2_	0.97 (0.92–1.01)	0.172		
CT90	0.99 (0.97–1.01)	0.350		

*AHI* apnea–hypopnea index, *AI* apnea index, *CI* confidence interval, *CT90* cumulative time percentage with SaO_2_ < 90%, *OR* odds ratio, *REM* rapid eye movement, *SaO*_*2*_ arterial oxygen saturation, *TST* total sleep time.

## Discussion

PAP is the first-line treatment modality for moderate-to-severe OSA. PAP works as a pneumatic splint by increasing the upper airway pressure during sleep. It maintains upper airway patency, thus effective in resolving as well as preventing upper airway collapse^16^. However, maximizing adherence to PAP is a major clinical obstacle as the treatment effect is interrelated with frequency and duration of PAP device use. According to a report which investigated the patterns of CPAP use of OSA patients, only 46% of patients met the criteria of regular users which was defined by administration of PAP device for at least 4 h in at least 70% of all nights^17^. Although adherence rate was increased after NHI coverage because the cost of PAP therapy is also one of the important factors for improving PAP adherence^9,18^, identifying the predictors of PAP adherence is important in managing OSA and lowering the risk of comorbidity. Furthermore, there are few identified PSG variables which can be used to reliably predict the PAP adherence. Therefore, this study was designed to investigate the possible polysomnographic parameters to identify OSA patients with good adherence to PAP treatment under NHI coverage.

Adherence to PAP therapy is influenced by device-related factors and side effects, psychological factors and family support^18^. Device-related issues include difficulties in exhaling due to high pressure, trouble falling asleep, frequent awakening, sore eyes, dry nose or mouth, nasal congestion, air leak, skin marks, bothersome noise, air swallowing and feelings of claustrophobia^12,13^. These factors are known to be associated with poor adherence. In contrast, adherence can be significantly improved through systematic education, prompt troubleshooting, and high-quality service by physicians and device managers^9^. Various demographic and clinical predictors have yielded inconsistent results through many studies. Female gender, older age, comorbid hypertension, and reduced ESS scores had a considerable correlation with increased PAP use^15,19,20^. However, another studies showed that 18- to 30-year-old women had the lowest adherence and hypertension was a factor for poor PAP adherence^18,21,22^. Although some studies reported that the higher AHI, higher BMI, higher ESS, and lower AHI during PAP use were associated with long-term use of PAP^12,15,22^, other reports demonstrated that OSA severity had no significant impact on the PAP adherence^15,18^. Furthermore, it is also known that patients with positional OSA are less adherent to PAP therapy^23^.

In this study, the group of good adherence comprised of 121 patients and the group of poor adherence comprised of 37 patients, resulting in a total good adherence of 76.6%. There was no significant correlation with good adherence in regard to sex, age, BMI, history of hypertension, cardiac disease, neurovascular disease, diabetes, neck circumference, waist circumference, and ESS. In addition, we investigated possible predictive factors of PAP adherence based on PSG data. None of the polysomnographic variables such as TST, sleep efficiency, arousal index, percentage of sleep stages including N1, N2, N3, and REM, total AI, total AHI, proportion of apnea and hypopnea in supine position, supine AHI, lateral AHI, mean SaO_2_, lowest SaO_2_, and CT90 showed statistically significant differences between adherent and non-adherent groups. The inconsistency between the results of this study and those of the previous studies might have been due to the fact that we enrolled patients who were prescribed with PAP after NIH coverage from single center during the adaptation period for 90 days, which could increase PAP adherence.

The percentage of sleep time on back over TST was the only polysomnographic parameter that was significantly higher in the adherent group than the non-adherent group, and as the sleep time on back increased, adherence showed tendency to increase. The cut-off value was determined to be 41.45% by ROC curve analysis, which means the patients with a percentage of sleep time on back of 41.45% or more showed significantly higher adherence rate than those with less than 41.45%. Furthermore, we wanted to figure out whether this variable shows statistically significant value under the possible effects of other covariates. All the variables with multicollinearity were excluded. Potential confounders such as gender, age, BMI, and comorbidities of hypertension, cardiac disease, neurovascular disease, and diabetes, irrespective of statistical significance, were included in the multivariate analysis. The adjusted OR for the percentage of sleep time on back of 41.45% or more still showed statistically significant value of 2.97 (95% CI 1.31 to 6.77;* p* = 0.009). Therefore, the patients with percentage of sleep time on back of 41.45% or more had 2.97 times higher adherence compared to the patients with percentage of sleep time on back less than 41.45%. All these things considered, sleep time on back of 41.45% or more could be regarded as a factor that can be sufficiently referred to clinically.

Although prevalence and severity of OSA is likely to increase in supine position than non-supine position, sleeping on one’s back may be considered more beneficial than sleeping on one’s side to improve PAP adherence. While using PAP, patients must wear face masks such as full-face masks, nasal masks and nasal pillows, and the masks are connected to the PAP machine with a hose. Previous study showed that during the adaptation period, discomfort from mask was the predominant reason for quitting PAP therapy^9^. To prevent the air leaks, the PAP mask should fit snugly to the face while not being too tight to cause any pain or irritation. Side sleeping may cause mask movement during the night, leading to air leaks and eye irritation. Some masks also create uncomfortable pressure on the cheek. Therefore, the more sleep time on back, the higher mask compliance, and PAP adherence improves.

This study has some limitations. First of all, the definition of good adherence was only concerning the first 90 days of PAP use and the data were collected from a single center with small sample size during a limited period of time. Therefore, the results cannot be generalized to all OSA patients. Furthermore, because it is not easier than usual for patients to change their posture during the PSG due to various sensors or a changed sleeping environment and sleep time on the back can vary from one day to the other, it cannot be guaranteed that one time PSG reflects the actual sleeping pattern. Although this study was intended to find factors that can predict adherence to some extent only with the PSG results before performing PAP, more clinically meaningful values can be obtained by analyzing the sleeping position and adherence while wearing PAP. Further study is required to evaluate the anatomical features in the pharynx or additional factors that may have an influence on PAP adherence or sleeping positions during PAP application. Moreover, prospective study with devices that prevent certain sleeping position could give us much insight about the relationship between sleeping position and PAP adherence. The predictors of long-term PAP adherence can be investigated as these results continue to be followed closely over time.

## Conclusion

The percentage of sleep time on back of 41.45% or more had an adjusted OR of 2.97 to be associated with good adherence in the first 90 days of PAP use. The percentage of sleep time on back was the only polysomnographic predictor for identifying the good adherence to PAP therapy in OSA patients.

## Data Availability

The datasets generated and/or analyzed during the current study are available from the corresponding author on reasonable request.
